# A Machine Learning-Based Prediction Model for Cardiovascular Risk in Women With Preeclampsia

**DOI:** 10.3389/fcvm.2021.736491

**Published:** 2021-10-27

**Authors:** Guan Wang, Yanbo Zhang, Sijin Li, Jun Zhang, Dongkui Jiang, Xiuzhen Li, Yulin Li, Jie Du

**Affiliations:** ^1^Beijing Anzhen Hospital, Capital Medical University, The Key Laboratory of Remodeling-Related Cardiovascular Diseases, Ministry of Education, Beijing Institute of Heart, Lung and Blood Vessel Diseases, Beijing, China; ^2^Beijing University of Chinese Medicine Third Affiliated Hospital, Beijing, China; ^3^Department of Health Statistics, School of Public Health, Shanxi Medical University, Shanxi Key Laboratory of Major Diseases Risk Assessment, Taiyuan, China; ^4^First Hospital of Shanxi Medical University, Molecular Imaging Precision Medicine Collaborative Innovation Center, Shanxi Medical University, Taiyuan, China

**Keywords:** preeclampsia, hypertension, cardiovascular disease, machine learning, prediction, model

## Abstract

**Objective:** Preeclampsia affects 2–8% of women and doubles the risk of cardiovascular disease in women after preeclampsia. This study aimed to develop a model based on machine learning to predict postpartum cardiovascular risk in preeclamptic women.

**Methods:** Collecting demographic characteristics and clinical serum markers associated with preeclampsia during pregnancy of 907 preeclamptic women retrospectively, we predicted the cardiovascular risk (ischemic heart disease, ischemic cerebrovascular disease, peripheral vascular disease, chronic kidney disease, metabolic system disease or arterial hypertension). The study samples were divided into training sets and test sets randomly in the ratio of 8:2. The prediction model was developed by 5 different machine learning algorithms, including Random Forest. 10-fold cross-validation was performed on the training set, and the performance of the model was evaluated on the test set.

**Results:** Cardiovascular disease risk occurred in 186 (20.5%) of these women. By weighing area under the curve (AUC), the Random Forest algorithm presented the best performance (AUC = 0.711[95%CI: 0.697–0.726]) and was adopted in the feature selection and the establishment of the prediction model. The most important variables in Random Forest algorithm included the systolic blood pressure, Urea nitrogen, neutrophil count, glucose, and D-Dimer. Random Forest algorithm was well calibrated (Brier score = 0.133) in the test group, and obtained the highest net benefit in the decision curve analysis.

**Conclusion:** Based on the general situation of patients and clinical variables, a new machine learning algorithm was developed and verified for the individualized prediction of cardiovascular risk in post-preeclamptic women.

## Introduction

Cardiovascular disease (CVD) is the leading cause of death among women, accounting for one-third of all women's deaths in the world (1). In the last decade, the mortality rate of CVD among young women has increased (1). Preeclampsia is the most serious form of hypertensive disorder of pregnancy. As a gender-specific risk factor for CVD, preeclampsia occurs in 2–8% of pregnant women worldwide and doubles the risk of CVD (2).

Studies have found that preeclampsia and CVD have common risk factors, such as obesity, hypertension, inflammation (3), while preeclampsia may also cause long-term changes in blood vessels and metabolism, thus increasing the risk of postpartum CVD. Besides, preeclampsia is considered as a failed stress test (4), which can be used to identify women with potential CVD (5), and then an appropriate intervention can be timely provided through targeted prevention of CVD. Current guidelines recommended that clinicians should screen women for pregnancy complications and monitor postpartum risk factors of CVD (6, 7).

Risk prediction tools can be employed to identify individuals with high CVD risks, so that targeted intervention and prevention can be performed to maximize the benefits of patients. However, ACC/AHA (1) have pointed out that the young women with a higher risk of CVD (8) are rarely targeted by the current tools. The calculation of 10-yr CVD incidence in young women may significantly underestimate the life time incidence of CVD. Therefore, early detection of subclinical CVD after preeclampsia is more important for CVD prevention among women with a history of preeclampsia. To this end, a verified tool is urgently required to screen out high-risk preeclamptic women with postpartum CVD and perform a targeted intervention on their risk factors. This tool is significantly helpful for personalized management of the cardiovascular risk of women after preeclampsia.

Machine learning (ML) is considered as an objective and reproducible method for integrating multiple quantitative variables to improve the diagnostic accuracy (9). In population studies, ML may be used to effectively characterize the cardiovascular risk, predicting the outcomes, and identify the biomarkers without a priori assumption of causation.

In this study, an ML algorithm suitable for most medical institutions was developed to predict the postpartum CVD risks of preeclamptic women by the ready-made clinical variables.

## Methods

This study is reported according to the Transparent Reporting of a multivariable prediction model for Individual Prognosis or Diagnosis(TRIPOD) statement (10) (Supplementary Table 1).

### Data Sources and Study Population

From January 2010 to April 2019, 1,063 hospitalized women diagnosed as having preeclampsia in the Obstetrics Department of Beijing Anzhen Hospital, Capital Medical University were collected. Women diagnosed as having preeclampsia were included in the study, as defined by the International Society for the Study of Hypertension in Pregnancy (ISSHP) 2018 Guideline (11). The management for preeclamptic women, preliminary assessment and continuous monitoring of the disease were in line with ISSHP Guideline (11, 12).

**Figure 1:**
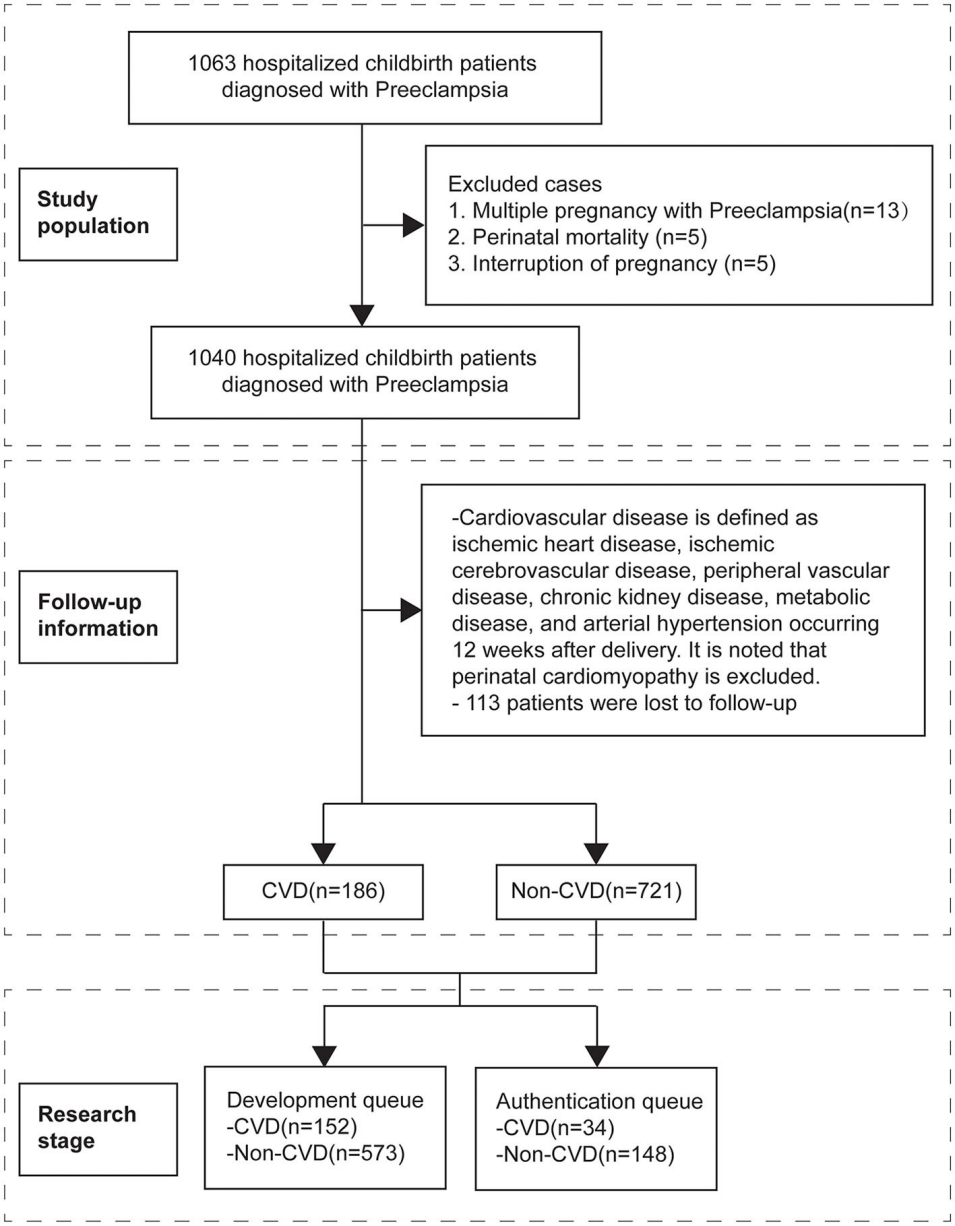
Study flow. CVD, Cardiovascular disease.

For women with multiple-preeclampsia history, the first preeclampsia pregnancy was selected. Women were excluded if they: (1) died in the perinatal period; or (2) were lost to follow-up; (3) had termination of pregnancy without indications for delivery; (4) had any Cardiovascular endpoint within 12 wk postpartum; or (5) had perinatal CVD (e.g. perinatal cardiomyopathy). Finally, 907 cases were enrolled (Figure 1).

### Candidate Predictor

In our study, the predictive, available, measurable, frequent and reliable variables were selected as candidate predictive variables (Table 1). Clinical predictive variables were set to the worst value within the 24 h before or after preeclampsia diagnosis. Although the performance of the model may be overestimated, the severity of preeclampsia can be directly reflected. The gestational age of the preeclamptic women was estimated based on the women's last menstruation and fetal ultrasound data. None of the pregnant women included in the study had a history of smoking.

**Table 1:** Comparison of participant characteristics by cardiovascular disease endpoints.

	**Women with CVD (*n* = 186)**	**Women without CVD (*n* = 721)**	**Number with missing data, *n* (%)**	***p* value**
**Demographic characteristics**				
Age, y	31.0 [28.0, 34.0]	30.0 [28.0, 34.0]	0	0.385
Gestational week at delivery, w	37.0 [32.0, 38.0]	38.0 [35.0, 39.0]	0	<0.001
^*^Multifetation, *n* (%)	11 (5.90)	57 (7.90)	0	0.358
^†^Multiple pregnancy, *n* (%)	107 (57.5)	382 (53.0)	0	0.268
^‡^Parity, *n* (%)	143 (76.9)	562 (77.9)	0	0.755
Gestational diabetes mellitus, *n* (%)	46 (24.7)	157 (21.8)	0	0.389
**Pre-pregnancy risk factor**				
Hypertension, *n* (%)	42 (22.6)	122 (16.9)	0	0.074
Diabetes mellitus, *n* (%)	8 (4.30)	25 (3.50)	0	0.588
Body mass index, kg/m^2^	24.2 [21.1, 26.6]	23.7 [20.5, 27.0]	0	0.985
**Blood pressure**				
Systolic BP at eligibility, mmHg	160 [141, 180]	146 [131, 160]	0	<0.001
Diastolic BP at eligibility, mmHg	100 [90, 110]	90 [80, 101]	0	<0.001
**Laboratory data**				
platelet count, (× 10^9^ /L)	236 [195, 291]	230 [193, 272]	0	0.032
neutrophil count, (× 10^9^/L)	11.3 [8.04, 14.0]	9.53 [7.36, 12.1]	0	<0.001
monocyte count, (× 10^9^/L)	0.69 [0.53, 0.88]	0.60 [0.47, 0.78]	0	0.002
Hemoglobin, g/L	124.5 [115.0, 135.0]	123.0 [115.0, 133.0]	0	0.142
Red blood cell specific volume, (%)	38.0 [35.0, 41.0]	37.1 [34.9, 40.0]	0	0.022
Glucose, mmol/L	6.45 [5.13, 7.63]	5.67 [4.77, 6.78]	0	<0.001
Aspartate aminotransferase, U/L	20.0 [16.0,29.0]	20.0 [17.0, 27.0]	0	0.104
Alanine aminotransferase, U/L	13.0 [9.00, 20.0]	13.0 [9.00, 20.0]	0	0.191
Alkaline phosphatase, U/L	138.0 [97.3, 185.0]	153.0 [114.0, 197.0]	16 (1.8)	0.027
Urea nitrogen, mmol/L	5.30 [4.00, 7.40]	4.40 [3.60, 5.70]	0	<0.001
Uric acid, μmol/L	391.6 [311.1, 484.4]	347.7 [276.7, 440.0]	0	<0.001
Creatinine, μmol/L	62.1 [50.8, 76.6]	56.7 [48.6, 67.5]	0	<0.001
24h proteinuria, mg	922.2 [406.7, 4101.3]	551.3 [354.2, 1667.9]	0	<0.001
Lactate dehydrogenase, U/L	212.0 [170.3,304.8]	197.0 [165.0, 259.0]	44 (4.9)	0.015
C-reactive protein, mg/l	4.95 [2.34, 10.69]	3.64 [1.76, 9.13]	143 (15.8)	0.110
Type B natriuretic peptide	181.5 [63.8, 430.8]	128.0 [50.0,353.5]	295 (32.5)	0.145
Homocysteine, umol/l	6.80 [5.70, 8.80]	6.90 [5.80, 8.60]	272 (30.0)	0.577
D-Dimer, ng/ml	870.0 [538.0,2423.0]	750.0 [444.5, 1882.0]	157 (17.3)	0.114
Fibrinogen, g/L	4.42 [3.89, 4.96]	4.33 [3.80, 4.83]	157 (17.3)	0.470
**Interventions**, ***n*** **(%)**	109 (58.6)	406 (56.3)	0	0.574
**Umbilical artery systolic/diastolic ratio**	2.50 [2.30, 2.90]	2.40 [2.20, 2.70]	29 (3.2)	0.061

*Values are interquartile range*.

* *Multifetation was defined as multiple births gestation*.

† *Multiple pregnancy was defined as the number of pregnancies ≥2 times*.

‡ *Parity was defined as no previous births. Interventions, antihypertensive medications administered and/or MgSO4 administered; Umbilical artery systolic/diastolic ratio, the ratio of peak systolic velocity of umbilical artery blood flow to minimum end-diastolic velocity (umbilical artery Doppler)*.

### Cardiovascular Endpoints

Based on extensive literature review (13–15) and experts' consensus (3, 4, 16–18), the composition of total CVD (the first attack of any cardiovascular or cerebrovascular disease and its risk factors) includes ischemic heart disease, ischemic cerebrovascular disease, peripheral vascular disease, chronic kidney disease, metabolic system disease (5) and arterial hypertension (Table 2). Metabolic system diseases are also considered as an important risk factor of CVD (5). If this risk factor is not included in cardiovascular endpoints, the real incidence rate of CVD will be underestimated and the actual risk prediction of CVD in the prognosis model will be affected. Patients whose cardiovascular events occurred within 12 wk after the end of pregnancy were excluded from the study, because these endpoint events were within the complication period for preeclampsia and could represent complications related to the pregnancy.

**Table 2:** Occurrence of cardiovascular endpoints in women following preeclampsia by morbidity event.

**Cardiovascular endpoints**		**Data**	**ICD-10**
**Total**		**186/907**	
Central Nervous System Diseases	Ischemic cerebrovascular disease	6	I63–I66, I69.3–I69.8
	Cerebral hemorrhage	1	I60–I62,I69.0–I69.2
Cardiovascular Disease	Ischemic heart disease	9	I20–I25
	Heart failure	8	I50, I11
	Arrhythmia	10	I44–I49
	Cardiomyopathies	1	I42
	Major artery aneurysms or dissections	2	I71–I72
Hypertension	Hypertension	84	I10–I13
Peripheral vascular disease	Thromboembolism	4	I80–I82
	Peripheral Atherosclerosis	3	I70
Chronic kidney disease	Renal glomerular disease	31	N00–05
	Renal insufficiency	6	N17–19
Metabolic diseases	Diabetes mellitus	9	E11, E14
	Dyslipidemia	21	E78

From 2010 to 2019, all women were followed up every two yr, with a median follow-up time of 70 months (6 months to 95 months). Follow-up time was calculated from time of index delivery, and women were followed until first the cardiovascular endpoints, whichever occurred first. The follow-up data were obtained by at least one of the following methods: records from the women's hospital visit, telephone interviews by the doctor who contacted the woman, and telephone interviews conducted by trained personnel during regular outpatient follow-up.

### Data Quality and Missing Data

In this study, data and diagnosis came from the women' electronic health record (EHR). Based on the International Classification of Diseases code (ICD)-10 for all outpatient and discharge diagnoses, outcome events of the patients were obtained; then these outcome events were medically verified by reviewing the medical records, the results of the relevant tests. The self-reported diagnoses were verified by medical records review or contact with the attending physician. Since diagnostic verification was based on the review and approval of medical instruments by a specialist, therefore, it is believed that the EHR data is highly accurate.

If data were missing, the method of the last observation was used, that is, any previous observation results recorded within one wk after diagnosis were considered as the latest observation results unless replaced by updated values. To this end, the data collection methods were checked, 100 cases (11.0%) of suspected or confirmed CVD were selected randomly, and the logic of the data was monitored.

The variables with missing values of >20% (Type B natriuretic peptide, Homocysteine) were removed. Then the chain equation was used for multiple imputations to estimate the missing values to avoid deviation, and 5 sets of imputation data were generated. A sensitivity analysis was performed using 5 sets of imputation data queue, and the distribution of each imputation data set was consistent with the original data. The sjmisc package merges 5 sets of imputation data into a plausible imputed data frame, which were used for all subsequent analyses (19).

### Machine Learning

The prediction model was developed by Logistic Regression (LR), Random Forest (RF), Support Vector Machines with Linear Kernel (SVM), Naive Bayes (NB), Extreme Gradient Boosting (XGBoost) algorithm. LR was used to predict the probability of events with discrete dependent variables. SVM was used to classify data points by maximizing the margin between classes. NB was a probability classifier with a strong assumption of independence between variables or features. RF was an integrated learning method in which multiple decision trees were constructed and averaged to form a solution to prevent over-fitting of training data. As a comprehensive decision tree method, Xgboost was designed to optimize the differential loss function. In our data, the loss function was the area under the curve (AUC).

**Figure 2:**
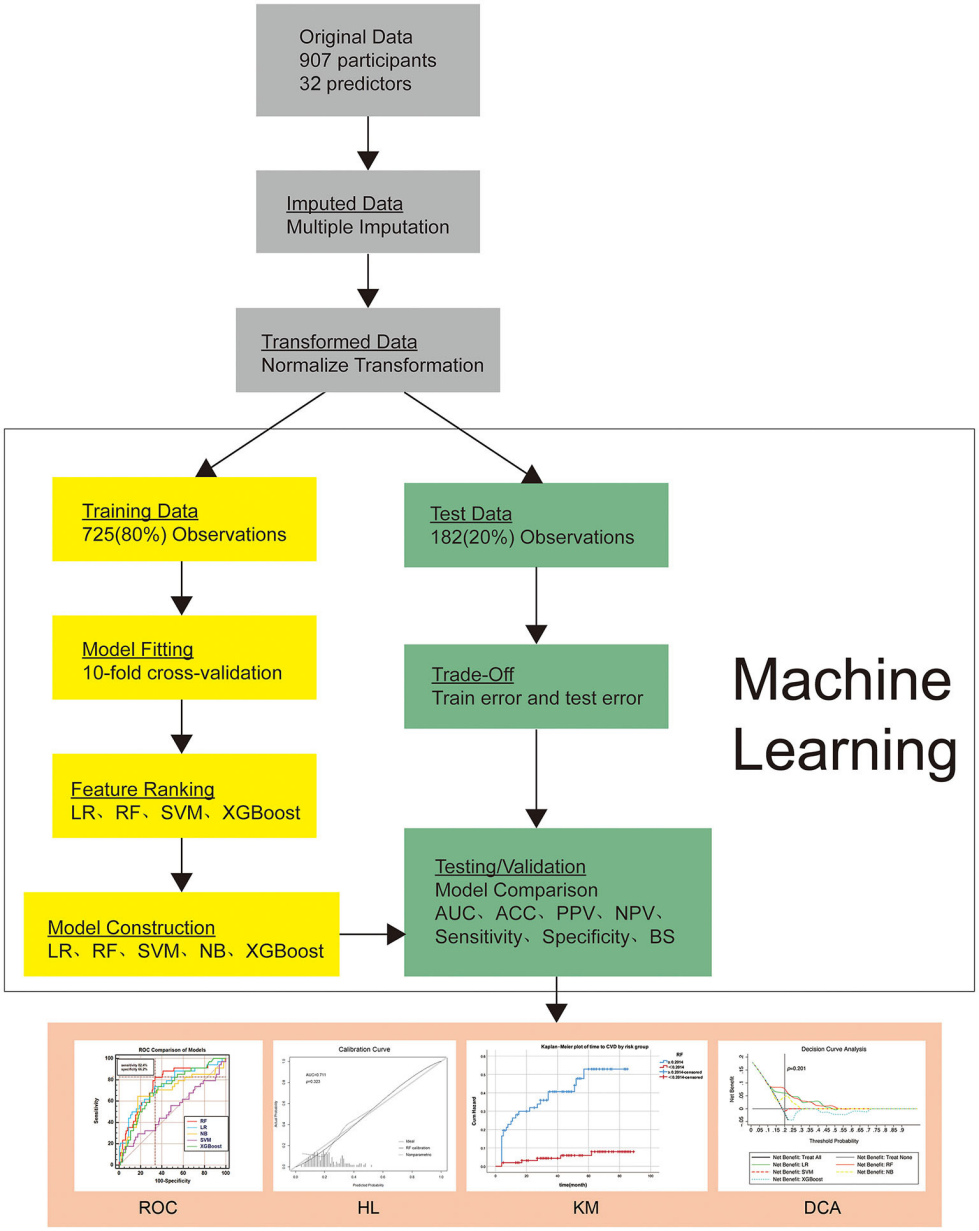
Overview of the methods used for data extraction, training, and testing. ROC, Comparison of ROC AUC of five machine learning algorithms; KM, Kaplan–Meier plot of time to CVD by risk group; DCA, Decision curve analysis curve for the five machine learning algorithms. HL, Hosmer-Lemeshow goodness-of-fit test of the Random Forest model in the validation set. DCA: The y-axis measures the net benefit. Gray line (Treat None) represents the net benefit of outcomes (postpartum CVD) of non-intervention for all pregnant women; Black line (Treat All) represents the net benefits of outcomes of intervention for all pregnant women; Lines in different colors represent the risk stratification of preeclamptic women according to different machine learning algorithms. When intervention is given to high-risk women, the net benefit of postpartum CVD risk may be generated. The model with the highest net income under a specific threshold has the highest clinical value. CVD, Cardiovascular disease; LR, Logistic Regression; RF, Random Forest; SVM, Support Vector Machines with Linear Kernel; NB, Naive Bayes; XGBoost, Extreme Gradient Boosting algorithm; AUC, area under the curve; ACC, accuracy; BS, Brier Score; PPV, positive predictive values; NPV, negative predictive values; ROC, receiver operating characteristic curve; KM, Kaplan–Meier curve; DCA, decision curve analysis.

Since the maximum values of variables in women after preeclampsia diagnosis were selected, all data were analyzed after normalization to reduce the impact of outliers. The study samples were divided into training sets and test sets randomly in the ratio of 8:2. In the process of training, the built-in function of ML algorithms was used in 5 ML algorithms to perform the 10-fold cross-validation with each training unit. Grid search was employed to select the optimal hyper-parameters value of ML algorithms. Key hyper-parameters and full details of model development can be found in the Supplementary Table 2. By weighing the best AUC, the final prediction model was established. In the test set, AUC, accuracy, Brier Score (BS), sensitivity, specificity, positive (PPV) and negative predictive values (NPV) were estimated respectively (Figure 2). To determine the optimal model and render the results more stable, the entire process was repeated 50 times, and the performance of the model was compared. Feature ranking was obtained by computing Shapley Additive Explanation values (SHAP), ML technology was implemented in Python 3.6 using the open-source scikit-learn (version 0.22.1) library.

## Statistical Analyses

As a supplementary method to solve the complexity of data set and expand the scope of the statistical model, ML analysis was performed on those data. The discrimination of ML algorithm was evaluated by measuring the total AUC, and the calibration of ML algorithm was evaluated by Hosmer-Lemeshow goodness-of-fit test and BS. The difference between the estimated risk of occurrence of CVD and the observed risk was calculated by BS (ranging from 0 to 1). In addition, SHAP values were presented to predict the individual postpartum CVD risk for each woman.

Receiver operating characteristic (ROC) curve was used to find the cut-off value of the predicted probability. The cut-off value is selected based on the probability threshold of Youden's index. The study population was divided into high-risk groups and low-risk groups based on cut-off value. The cumulative risk curve was drawn by Kaplan-Meier methods. Based on Kaplan-Meier analysis and log-rank test, we compared the cumulative risk incidence of between the two groups.

However, cut-off value is obtained theoretically. In clinical practice, pregnant women with preeclampsia are not simply predicted to have CVD, or pregnant women without preeclampsia are predicted to be free of CVD; the possibility of the net benefit of the model is determined over a wide range of threshold probabilities. We express the net benefit as a function of the decision threshold in the decision curve. The choice of the threshold depends on the intervention, clinician preference and patient situation. For example, if the intervention involves that pregnant women with preeclampsia are referred to a tertiary hospital for prenatal and intrapartum care, a lower threshold can be used to minimize false negatives and unnecessary referrals. If a higher threshold is chosen, fewer women are admitted to the hospital, while some false-negative women may lose admission for observation and tertiary care. However, if the proposed intervention aims to increase the dose and number of anti-hypertensive drug distributors (which can have maternal side effects and increase the risk of neurodevelopmental problems in the fetus later in life), then the pediatrician may choose a higher threshold than that in hospitalization. In addition to screening high-risk patients (e.g. eclampsia, severe hypertension), unnecessary interventions that may adversely affect the mother or the fetus must be considered in the decision-making principles. Finally, the clinical application value of the model was evaluated by decision curve analysis (DCA).

Continuous variables were compared using a two-tailed student *t-*test, while categorical data were analyzed using Chi-square or Fisher exact test. DCA was operated on the Stata (version 13.0; StataCorp), Statistical analysis was conducted by SPSS statistics 25.0. All statistical tests were two-tailed and *p* < 0.05 was considered significant.

## Results

### Clinical Features

From January 2010 to April 2019, 1,063 preeclampsia cases were collected. Among them, the follow-up of 113 cases was lost. Therefore, 907 patients were included in the study, and 186 (20.5%) had CVD. 148 preeclamptic women were followed up for one yr (16.3%). Ninety-five women had CVD within one yr, predominantly chronic hypertension which occurred in 51 women (20) (Supplementary Figure 1). Compared with patients without CVD, pregnant women with CVD were older and delivered earlier. The blood pressure, fasting glucose and markers of inflammation, coagulation, renal function after preeclampsia diagnosis were significantly higher than those of patients without CVD (Table 1) Baseline sociodemographic and clinical attributes were generally similar between those who were lost to follow-up and those remained in the sample (Supplementary Table 3).

The most common postpartum CVD risk in patients were hypertension (84 women [45.2%]), and dyslipidemia (21 women [11.3%]). Two women had acute cerebrovascular disease (1.1%) (Table 2).

### Development of Model

Five ML algorithms were used to develop the prediction model. In the modeling process, the 10-fold cross-validation optimization model was performed and applied to the test set, respectively. Then the performance of the five models was compared. Considering the data imbalance, the weighted-average parameter was used to weight the score of each class. The model obtained by RF has the best discrimination (AUC = 0.711; 95%CI 0.697–0.726) (Figure 2 and Table 3).

**Table 3:** Comparison of prediction results of five test models using test data sets.

	**LR**	**SVM**	**NB**	**xgBoost**	**RF**
**AUC**	0.678 (0.660–0.696)^*^	0.606 (0.594–0.617)^*^	0.629 (0.611–0.646)^*^	0.683 (0.670–0.696)^*^	0.711 (0.697–0.726)
**Accuracy**	0.810 (0.795–0.820)	0.799 (0.785–0.810)	0.801 (0.791–0.813)	0.803 (0.782–0.823)	0.817 (0.797–0.827)
**Sensitivity**	0.802 (0.793–0.811)	0.796 (0.785–0.806)	0.802 (0.792–0.813)	0.802 (0.792–0.813)	0.815 (0.795–0.825)
**Specificity**	0.994 (0.991–0.997)	0.981 (0.972–0.994)	0.987 (0.978–0.995)	0.977 (0.972–0.981)	0.984 (0.945–0.997)
**PPV**	0.779 (0.753–0.806)	0.634 (0.617–0.650)	0.725 (0.685–0.765)	0.771 (0.758–0.785)	0.777 (0.749–0.804)
**NPV**	0.804 (0.794–0.813)	0.796 (0.785–0.806)	0.797 (0.787–0.808)	0.812 (0.802–0.823)	0.807 (0.797–0.817)
**Brier Score**	0.150 (0.144–0.156)	0.161 (0.155–0.167)	0.158 (0.152–0.164)	0.163 (0.160–0.167)	0.133 (0.120–0.141)

*The sensitivity, specificity, accuracy, positive (PPV) and negative predictive values (NPV) are presented as %(simple counts). The area under the curve(AUC) is presented as AUC value(95% confidence interval)*.

* *P < 0.05 vs. RF, DeLong test. LR, Logistic Regression; RF, Random Forest; SVM, Support Vector Machines with Linear Kernel; NB, Naive Bayes; XGBoost, Extreme Gradient Boosting algorithm; AUC, area under the curve; BS, Brier Score; PPV, positive predictive values; NPV, negative predictive values*.

**Figure 3:**
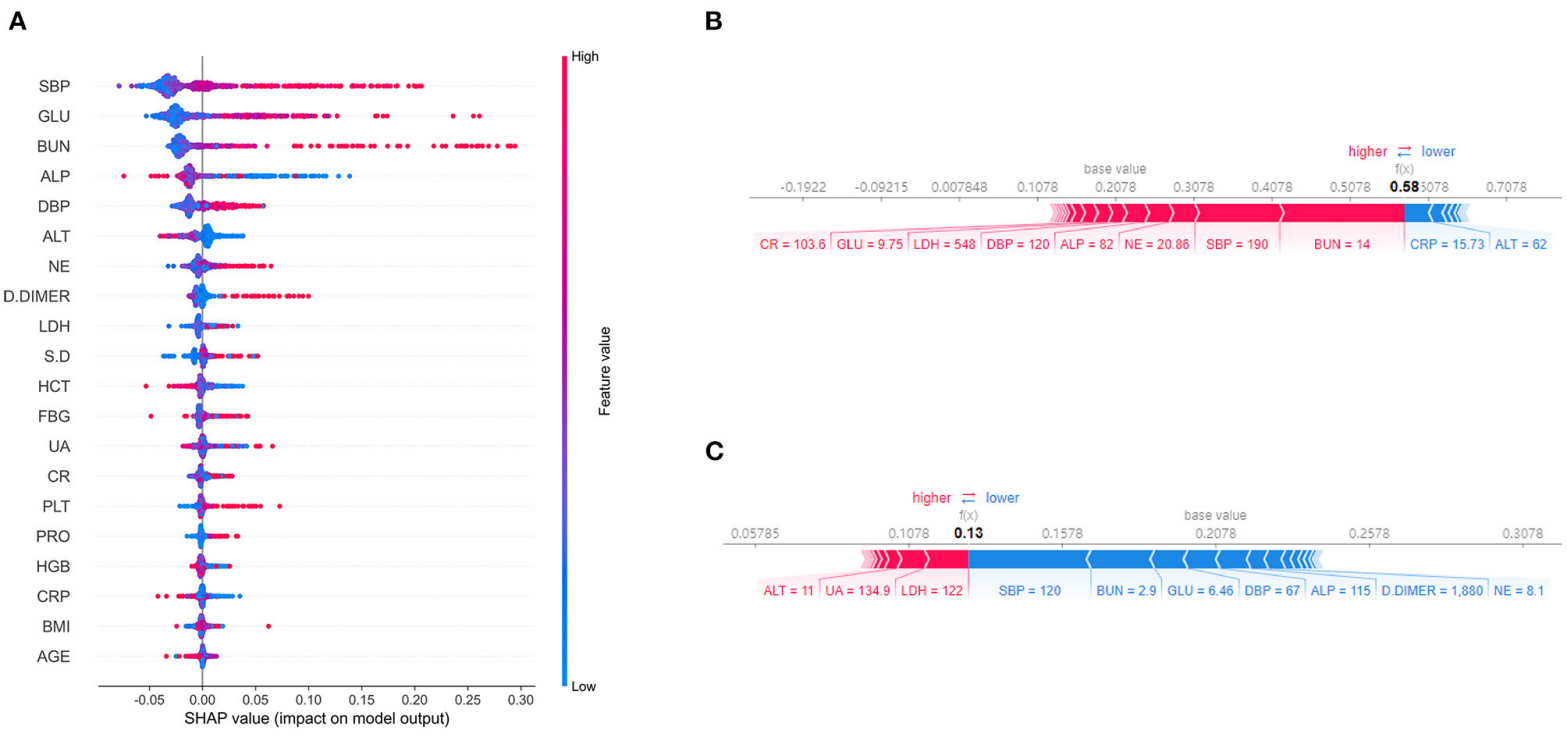
Feature importance plot for the machine learning model. **(A)** The top 20 clinical variables evaluated by the average absolute SHAP value. The red and blue points in each row represent preeclamptic women having high to low values of the specific predictor, while the x-axis gives the SHAP value which gives the effect on the model [i.e. does it tend to drive the predictions toward CVD (positive value of SHAP) or non-CVD (negative value of SHAP)]. **(B)** and **(C)** SHAP values to explain the predicted postpartum cardiovascular risk of two individuals. The baseline (the average predicted probability) is 0.2078. **(B)** The woman with CVD has a high predicted risk of 0.58. Risk increasing effects such as SBP = 190 and BUN = 14 increase her predicted postpartum cardiovascular risk. **(C)** The woman without CVD has a low predicted risk of 0.13. Risk increasing effects such as LDH = 122 are offset by decreasing effects such as SBP. SHAP, Shapley Additive Explanation values; SBP, systolic blood pressure; GLU, glucose; BUN, Urea nitrogen; ALP, Alkaline phosphatase; DBP, diastolic blood pressure; ALT, alanine aminotransferase; NE, neutrophil count; LDH,Lactate dehydrogenase; S.D, umbilical artery systolic/diastolic ratio; HCT, Red blood cell specific volume; FBG, Fibrinogen; UA, uric acid; CR, creatinine; PLT, platelet count; PRO, 24h proteinuria; HGB, Hemoglobin; CRP,C-reactive protein; BMI, body mass index.

RF algorithm was adopted in the process of feature selection and the establishment of the prediction model. Figure 3A displays the top 20 most important features in our model. According to the importance ranking of the average absolute SHAP value, the most important variables were also the most commonly used direct indicators for the assessment of blood pressure, fasting glucose, inflammation (neutrophil count),renal function (Urea nitrogen),and coagulation function(D-Dimer). Furthermore, SHAP values can effectively clarify and explain model predictions for individual patients (Figures 3B,C).

### Performance Evaluation

RF algorithm was used in the test set with an AUC of 0.711 (95% CI 0.697–0.726) (Figure 2). The sensitivity, specificity, PPV, NPV and accuracy of the model for predicting postpartum CVD were 81.5% (95%CI 79.5–82.5%), 98.4% (95%CI 94.5–99.7%), 77.7% (95%CI 74.9–80.4%), 80.7% (95%CI 79.7–81.7%) and 81.7% (95%CI 79.7–82.7%) respectively. The BS for the RF algorithm predicting CVD was 0.133 (95%CI 0.120–0.141), the Hosmer-Lemeshow test yielded a non-significant statistic (*p* = 0.223, Figure 2), indicating that the model predicted the probability of CVD and the observed probability of CVD fit well (Table 3).

### Clinical Use

Receiver operating characteristic (ROC) curve was used to find the cut-off value of the predicted probability, and the cut-off value was 20.1%, the sensitivity was 82.4%, and the specificity was 66.2%. The cut-off value is selected based on the probability threshold of Youden's index. The study population were divided into high-risk groups and low-risk groups based on cut-off value. Based on Kaplan-Meier analysis and log-rank test, there was a significant difference in the cumulative incidence of CVD risk between the two groups (*p* < 0.05, Figure 2). At 40 months postpartum in preeclamptic patients, the cumulative risk of CVD disease was 40% in the high-risk group (blue line) and less than 5% in the low-risk group (red line). At 60 months postpartum, the cumulative risk of CVD disease was more than 50% in the high-risk group (blue line) and less than 10% in the low-risk group (red line).

The DCA of 5 ML algorithms is shown in Figure 2. RF algorithms have a net benefit of predicted probability thresholds between 12% and 53%. Compared with other algorithms, the net benefit in this range has obvious superiority. When the cut-off value (*p* = 20.1%) was taken as the prediction probability, the net benefit of the RF algorithm was significantly higher than that of other algorithms.

## Discussion

In this study, the most important variable features were selected from a large number of demographic characteristics and simple clinical variables related to preeclampsia diagnosis by ML methods; then a new decision-making tool was trained and tested to predict the occurrence of CVD risk, so as to further investigate the increased CVD risk by preeclampsia. The algorithm was verified and effectively employed in clinical practice to improve the risk stratification. In this way, cardiologists, obstetricians and gynecologists and general practitioners can use the proposed algorithm to identify women with high-risk diseases and perform postpartum prevention.

ML has been proved to be a powerful prediction tool in the cardiovascular field (21–27). The accurate identification of high-risk patients who may benefit from intensified preventive measures is the main challenge of cardiovascular medicine, and the purpose of our study. At present, maternal health care has been more and more perfect. In our study, the proportion of clinical intervention in pregnancy increased to 56.8%. But, it was found that hypertension is still an important predictor of CVD, and pregnancy intervention does not occupy an important position in the model. There may be neglected risk factors in the development of CVD in post-preeclamptic women. To exclude the influence of pregnancy confounding factors, clinical variables were included as much as possible, and the pre-pregnancy BMI was adjusted. The ML may provide an improved alternative scheme by constantly integrating new data, automatically updating and recalibrating over time, so that a non-linear model can be found to predict specific personal risks, namely, accurate risk stratification.

Previous studies have identified the correlation between preeclampsia and CVD (4, 5, 28–31). However, significant correlation cannot be necessarily converted into good prediction performance. At present, related studies have found that CVD can be independently predicted by preeclampsia, but the inclusion of preeclampsia history showed little or no improvement in the prediction of cardiovascular risk. On the one hand, since different clinical variables related to preeclamptic women play different roles in the development process of CVD, and these variables should be analyzed one by one rather than explained in a general way. On the other hand, the relationship between preeclampsia and CVD is mediated to a large extent by the risk factors of CVD (4, 28). Therefore, as the predictive variables, clinical variables during onset of preeclampsia should be collected as much as possible; the role of these variables in the prediction of CVD risk should be analyzed, and the CVD risk factors should be included in the outcome event for study.

Compared with pregnant women with normal blood pressure, post-preeclamptic women have an average twice higher risk of CVD in their later years. The increased risk may be due to potential CVD tendencies, preeclampsia itself, or a combination of both. On the one hand, preeclampsia and CVD have common risk factors, including genetic factors, diabetes, and hypertension (3). On the other hand, in essence, the progression of CVD is the progression of atherosclerosis. Atherosclerosis begins with the injury of vascular endothelium, followed by the intervention of inflammatory cytokines. During the onset of preeclampsia, persistent vascular endothelial injury and inflammatory stress may activate atherosclerosis procedure, which in turn lead to long-term vascular and metabolic changes in patients (13, 28). Even if postpartum blood pressure has returned to normal, these seemingly healthy women may suffer adverse metabolic and vascular changes (28). Therefore, classic cardiovascular risk factors were increased for preeclamptic women after delivery, including chronic hypertension (5, 32), subclinical atherosclerosis, diabetes and renal insufficiency. Our study also found that correlated variables reflecting endothelial injury (blood pressure, Urea nitrogen), inflammation (neutrophil count), metabolic disorder(glucose) (33), coagulation abnormalities (D-Dimer) have important weights in predicting the occurrence of CVD. This may be because our CVD endpoint is a stage in the development of CVD, and serological indicators reflecting different aspects of CVD pathogenesis are important predictors of our CVD endpoint.

### Strengths

The most important value of the proposed model in the clinic is to help doctors decide the time of delivery. The risk prediction performance (discrimination and calibration) of the model can not reflect the clinical consequences of specific discrimination or incorrect calibration (10, 34, 35). The improvement in the patient's prognosis after the decision-making of clinical intervention based on the proposed model was evaluated to verify the clinical significance of the proposed model. For this reason, DCA was applied in this study, and multi-agency prospective external verification was no longer conducted. This method provides an evaluation perspective of clinical results based on threshold probability, from which net benefits can be obtained (10). The decision curve showed that if the threshold probability of patients or doctors was >20.1%, compared with other treatment options, using the model in the current study to predict the postpartum cardiovascular risk of preeclampsia significantly increased the benefits of patients.

Second, in order to optimize the prevention of CVD, our study suggested that screening and intervention of CVD should be started after preeclampsia diagnosis (4). Follow-up of CVD after delivery can be carried out by different health care professionals (including general practitioners, cardiologists or obstetricians and gynecologists) (3), so that the future risk probability of CVD in preeclamptic women was calculated, and personalized postpartum cardiovascular risk management for preeclamptic women can be formulated according to predictive factors.

### Limitations

Nevertheless, there are still some limitations in this study. Firstly, our study was a single center study, and the missing data were estimated in this study. Although corresponding statistical methods were used to minimize the over-fitting and our main results were supported by various analysis methods, many external validations for different settings and populations of the model was still needed to fully understand the transportability of the model (36). Secondly, the trade-off between simpler predictors and more comprehensive ML algorithms needs to be further evaluated. Thirdly, the sensitivity and specificity were 81.5 and 98.4% respectively. Our study had a good effect on excluding negative patients, but a slightly poor recognition ability of positive patients. It is referred that our outcome events contain a large number of CVD risk factors, and the risk factors of CVD are also affected by uncontrollable factors such as lifestyle. Therefore, more sensitive markers should be added to improve the prediction accuracy of the model. Finally, longer follow-up is required. Since the CVD risk in patients with preeclampsia within 1–9 yr after delivery is explored in this study, not the evaluation time point of CVD, longer follow-up is required to predict the occurrence of CVD. In a word, future research should be performed to verify the feasibility of the proposed algorithm.

## Conclusion

Based on readily available clinical and demographic variables, an ML algorithm was proposed to predict the CVD occurrence of post-preeclamptic women in this study. The proposed ML algorithm can be directly applied to clinical practice for the accurate identification of high-risk patients, and it can be taken as a convenient prediction tool for clinicians.

## Data Availability Statement

The original contributions presented in the study are included in the article/Supplementary Material, further inquiries can be directed to the corresponding authors.

## Author Contributions

GW designed the experiments, conducted the experiments, analyzed the data, and wrote the manuscript. JD, YZ, and YL designed the experiments and edited the manuscript. YZ did the data management and analysis. SL provided clinical input and reviewed the manuscript. JZ, DJ, and XL provided clinical input at all stages of the project. DJ and XL collected the trial data. All authors contributed to manuscript revision, read and approved the submitted version.

## Funding

This research was supported by a grant from the National Natural Science Foundation of China (Grant No: 82173631).

## Conflict of Interest

The authors declare that the research was conducted in the absence of any commercial or financial relationships that could be construed as a potential conflict of interest.

## Publisher's Note

All claims expressed in this article are solely those of the authors and do not necessarily represent those of their affiliated organizations, or those of the publisher, the editors and the reviewers. Any product that may be evaluated in this article, or claim that may be made by its manufacturer, is not guaranteed or endorsed by the publisher.
