# Supplementary material for: A Machine Learning-Based Prediction Model for Cardiovascular Risk in Women With Preeclampsia
Source: Front Cardiovasc Med. 2021 Oct 27;8:736491. doi: 10.3389/fcvm.2021.736491 (PMC8578855; doi:10.3389/fcvm.2021.736491)
Supplement: Supplementary file 1 [file Presentation_1.zip › Supplementary_Material/Supplementary_Material.docx]

Supplementary Material

**Supplementary Method**

# Supplementary Method 1. Modeling of the five machine learning algorithms

The prediction model was developed by Logistic Regression (LR), Random Forest (RF), Support Vector Machines with Linear Kernel (SVM), Naive Bayes (NB), Extreme Gradient Boosting (XGBoost) algorithm. In this study, a separate test set (not included in the training of the model) is used to evaluate the model and calculate different accuracy measures, with special attention to the AUC value. For all cases, the supervised learning classification algorithm in Python software is employed to implement each set of variables or features. The parameters of these algorithms are

illustrated in **Supplementary Table 3**.

**Logistic Regression**

LR is used to predict the probability of an event with a discrete dependent variable (1). In this model, a sigmoid function is used to predict the logical conversion of each type of probability in the dependent variable. The recorded probability classifies the data points as binary. In addition to the conjugate gradient descent, the parameter λ used in the model also has a ridge value of 1.0E-8 offspring. Conjugate gradient descent is applied to reduce the cost function in the model.

**Support Vector Machines with Linear Kernel**

In recent years, SVM has been introduced to solve various biomedical problems. As a supervised machine learning method, SVM is used to classify data points by maximizing the margin between classes(2). It can be used for nonlinear classification using kernel tricks. Besides, different kernel functions are used to map inputs implicitly to higher-dimensional feature Spaces. In this study, the radial basis function (RBF) kernel function is used. The recursive feature elimination method is applied to feature selection using training data sets. Subsequently, a random search strategy is implemented to adjust the parameters of the classifier, and a 10-fold cross-validation method is used during training. The parameters C and γ are determined to be 10 and 0.001, respectively.

**Naive Bayes**

Based on the Bayes theorem, Naive Bayes (NB) is a probabilistic classifier with a strong assumption of independence among variables or features (3). The result can be interpreted as a Bayesian network where nodes represent features that are interconnected by directed arcs. The model outputs the probability that each feature belongs independently to a particular category and takes into account all of the features to determine the category. Similar to the previous algorithm, we use the Weka software to train the NB algorithm with our training set. If batch forecasting is performed, the default parameter “batchSize” is used, which contains the number of preferred instances to process.

**Random forest**

RF is a classification algorithm that combines decision trees and bootstrap to collect training data and split branches in each tree (4). The goal of any partition is to maximize the information obtained from each random feature in each sample of each tree. After evaluating the data points, the resulting class is the pattern of the results for all trees. The algorithm is deployed in our dataset, Gini standards are used for splitting without pruning, and the int (log_2 (#predictors) t1) functions are used in every split of each tree. As a result, a total of 50 iterations of the model is performed, the sample is divided into training (80%) and testing (20%) subsets in these iterations.

**Extreme Gradient Boosting algorithm**

XGBoost is a gradient descent boosting method based on a series of decision trees for prediction (5). Tree-based modeling methods can adaptively learn higher-order interactions and explain non-linear relationships without pre-defined assumptions. Therefore, this method is more effective in capturing potentially complex relationships between variables and results. In this study, the original data set is randomly divided into a training set (80%) and a persistent validation set (20%). Then the training set is divided into 10 folds of equal size to roughly maintain the ratio of events to non-events in the training set. The optimal model hyperparameters are selected by grid search through 10 cross-validation. On the training set, the 10-fold cross-validation is used to fine-tune model hyperparameters.

**Supplementary Tables**

**Supplementary Table 1. TRIPOD Checklist: Prediction Model Development and Validation**

| **Section/Topic** | **Ite** |  | **Checklist Item** | **Page** |
| --- | --- | --- | --- | --- |
| **Title and abstract** | | | | |
| Title | 1 | D;V | Identify the study as developing and/or validating a multivariable prediction model, the target population, and the outcome to be predicted. | Title |
| Abstract | 2 | D;V | Provide a summary of objectives, study design, setting, participants, sample size, predictors, outcome, statistical analysis, results, and conclusions. | Abstract |
| **Introduction** | | | | |
| Background and objectives | 3a | D;V | Explain the medical context (including whether diagnostic or prognostic) and rationale for developing or validating the multivariable prediction model, including references to existing models. | Introduction, para 1-4 |
|  | 3b | D;V | Specify the objectives, including whether the study describes the development or validation of the model or both. | Introduction, para 5 |
| **Methods** | | | | |
| Source of data | 4a | D;V | - Describe the study design or source of data (e.g., randomized trial, cohort, or registry data), separately for the development and validation data sets, if applicable. | Methods,  para 2 and 3 |
|  | 4b | D;V | Specify the key study dates, including start of accrual; end of accrual; and, if applicable, end of follow-up. | Methods,  para 2 and 6 |
| Participants | 5a | D;V | - Specify key elements of the study setting (e.g., primary care, secondary care, general population) including number and location of centres. | Methods,  para 2 |
|  | 5b | D;V | - Describe eligibility criteria for participants. | Methods,  para 2 and 3 |
|  | 5c | D;V | - Give details of treatments received, if relevant. | Table1, Interventions |
| Outcome | 6a | D;V | - Clearly define the outcome that is predicted by the prediction model, including how and when assessed. | Methods, para5-7 and Table2 |
|  | 6b | D;V | - Report any actions to blind assessment of the outcome to be predicted. | Methods,  para 6 |
| Predictors | 7a | D;V | - Clearly define all predictors used in developing or validating the multivariable prediction model, including how and when they were measured. | Methods, para4 and Table1 |
|  | 7b | D;V | - Report any actions to blind assessment of predictors for the outcome and other predictors. | NA |
| Sample size | 8 | D;V | - Explain how the study size was arrived at. | NA |
| Missing data | 9 | D;V | - Describe how missing data were handled (e.g., complete-case analysis, single imputation, multiple imputation) with details of any imputation method. | Methods,  para 8 and 9 |
| Statistical analysis methods | 10a | D | - Describe how predictors were handled in the analyses. | Methods,  para 11 |
|  | 10b | D | - Specify type of model, all model-building procedures (including any predictor selection), and method for internal validation. | Methods,  para 11 |
|  | 10c | V | - For validation, describe how the predictions were calculated. | Methods,  para 11 |
|  | 10d | D;V | - Specify all measures used to assess model performance and, if relevant, to compare multiple models. | Methods,  para 11 and Table1 |
|  | 10e | V | - Describe any model updating (e.g., recalibration) arising from the validation, if done. | NA |
| Risk groups | 11 | D;V | Provide details on how risk groups were created, if done. | statistic analysis,  para 1 |
| Development vs. validation | 12 | V | For validation, identify any differences from the development data in setting, eligibility criteria, outcome, and predictors. | NA |
| **Results** | | | | |
| Participants | 13a | D;V | - Describe the flow of participants through the study, including the number of participants with and without the outcome and, if applicable, a summary of the follow-up time. A diagram may be helpful. | Results, Table1 and Figure1 |
|  | 13b | D;V | - Describe the characteristics of the participants (basic demographics, clinical features, available predictors), including the number of participants with missing data for predictors and outcome. | Results, para 1 and Table 1 |
|  | 13c | V | - For validation, show a comparison with the development data of the distribution of important variables (demographics, predictors and outcome). | Results, Table 1 and 2 |
| Model development | 14a | D | - Specify the number of participants and outcome events in each analysis. | Results, para 2 and Figure2 |
|  | 14b | D | - If done, report the unadjusted association between each candidate predictor and outcome. | NA |
| Model specification | 15a | D | - Present the full prediction model to allow predictions for individuals (i.e., all regression coefficients, and model intercept or baseline survival at a given time point). | Results, para 3 and Figure2 |
|  | 15b | D | - Explain how to the use the prediction model. | Results, para 4 |
| Model performance | 16 | D;V | - Report performance measures (with CIs) for the prediction model. | Results, Table 3 and para 5-7 |
| Model-updating | 17 | V | If done, report the results from any model updating (i.e., model specification, model performance). | Results, Table 3 and para 5-7 |
| **Discussion** | | | | |
| Limitations | 18 | D;V | Discuss any limitations of the study (such as nonrepresentative sample, few events per predictor, missing data). | Discussion, para 7 |
| Interpretation | 19a | V | For validation, discuss the results with reference to performance in the development data, and any other validation data. | Discussion, para 4 |
|  | 19b | D;V | Give an overall interpretation of the results, considering objectives, limitations, results from similar studies, and other relevant evidence. | Discussion, para 1-3 |
| Implications | 20 | D;V | Discuss the potential clinical use of the model and implications for future research. | Discussion, para 5-6 |
| **Other information** | | | | |
| Supplementary information | 21 | D;V | Provide information about the availability of supplementary resources, such as study protocol, Web calculator, and data sets. | Supplementary |
| Funding | 22 | D;V | Give the source of funding and the role of the funders for the present study. | NA |

**Supplementary Table 2. Comparison of characteristics of lost-to-follow-up and participant women**

|  | | Lost-to-follow-up Women (n=113) | participant Women (n=907) | *p* value | |
| --- | --- | --- | --- | --- | --- |
| Demographic characteristics | |  |  |  |  |
| Age, y | | 30.5 [27.0,34.0] | 30.0 [28.0,34.0] | 0.385 |  |
| Gestational week at delivery, w | | 37.0 [33.0,39.0] | 37.0 [35.0,39.0] | 0.472 |  |
| Multifetation, n(%) | | 8.0 (7.50) | 68.0 (7.50) | 0.989 |  |
| Multiple pregnancy, n(%) | | 59.0 (52.2) | 489.0 (53.9) | 0.717 |  |
| Parity, n(%) | | 89.0 (79.1) | 705.0 (77.7) | 0.720 |  |
| Gestational diabetes mellitus, n(%) | | 30.0 (26.9) | 203.0 (22.4) | 0.249 |  |
| Pre-pregnancy risk factor | |  |  |  |  |
| Hypertension, n(%) | | 23.0 (20.1) | 164.0 (18.1) | 0.564 |  |
| Diabetes mellitus, n(%) | | 4.00 (3.70) | 33.0 (3.60) | 1.000 |  |
| Body mass index, kg/m^2^ | | 23.6 [20.6, 27.1] | 23.8 [20.6, 27.0] | 0.847 |  |
| Blood pressure | |  |  |  |  |
| Systolic BP at eligibility, mmHg | | 144[125, 160] | 148[134, 161] | 0.099 |  |
| Diastolic BP at eligibility, mmHg | | 92[80, 103] | 92[82, 103] | 0.218 |  |
| Laboratory data | |  |  |  |  |
| platelet count, (×10⁹ /L) | | 234[192, 271] | 231 [192, 274] | 0.096 |  |
| neutrophil count, (×10⁹/L) | | 9.86 [7.43, 12.35 | 9.78 [7.49, 12.75] | 0.641 |  |
| monocyte count, (×10⁹/L) | | 0.65 [0.49, 0.82] | 0.62 [0.48, 0.80] | 0.079 |  |
| Hemoglobin, g/L | | 123.5 [114.0, 132.0] | 124.0 [115.0, 133.0] | 0.096 |  |
| Red blood cell specific volume,(%) | | 37.1 [34.8, 39.8] | 37.2 [34.9, 40.0] | 0.063 |  |
| Glucose, mmol/L | | 5.58 [4.76, 7.03] | 5.78 [4.82, 7.10] | 0.247 |  |
| Aspartate aminotransferase, U/L | | 19.5[15.8,28.0] | 20.0 [17.0, 28.0] | 0.333 |  |
| Alanine aminotransferase, U/L | | 13.0 [8.00, 19.3] | 13.0 [9.00, 20.0] | 0.237 |  |
| Alkaline phosphatase, U/L | | 162.0 [112.0, 207.0] | 150.0 [111.0, 194.0] | 0.260 |  |
| Urea nitrogen, mmol/L | | 4.60 [3.60, 6.10] | 4.60 [3.60, 5.90] | 0.108 |  |
| Uric acid, μmol/L | | 361.5 [263.5.1, 460.2] | 356.4 [283.3, 453.3] | 0.557 |  |
| Creatinine, μmol/L | | 55.9 [46.0, 67.6] | 58.0 [49.0 69.1] | 0.738 |  |
| 24h proteinuria, mg | | 622.8 [387.7, 2463.6] | 576.5[362.0, 1916.3] | 0.536 |  |
| Lactate dehydrogenase, U/L | | 232.5[187.0, 288.8] | 201.0 [166.0, 265.0] | 0.015 |  |
| C-reactive protein, mg/l | | 3.95 [1.95, 9.69] | 3.89[1.85, 9.33] | 0.196 |  |
| Type B natriuretic peptide | | 132.5[53.8,376.8] | 133.5[51.3,385.0] | 0.205 |  |
| Homocysteine, umol/l | 6.75 [5.78, 8.31] | | 6.90 [5.80, 8.60] | 0.351 |  |
| D-Dimer, ng/ml | | 870.0[538.0,2423.0] | 750.0[444.5, 1882.0] | 0.625 |  |
| Fibrinogen, g/L | | 4.24[3.77, 4.94] | 4.34 [3.82, 4.89] | 0.547 |  |
| Interventions , n(%) | | 65(57.9) | 515(56.8) | 0.302 |  |
| Umbilical artery systolic/ diastolic ratio | | 2.43[2.20, 2.80] | 2.40 [2.20, 2.70] | 0.349 |  |

Values are interquartile range.

Interventions, antihypertensive medications administered and/or MgSO4 administered; Umbilical artery systolic/ diastolic ratio, the ratio of peak systolic velocity of umbilical artery blood flow to minimum end-diastolic velocity (umbilical artery Doppler).

**Supplementary Table 3. Key hyperparameters used in different models**

| **Model** | **Parameter optimization range** | **Hyperparameters and corresponding values** |
| --- | --- | --- |
| **LR** | penalty':['l1', 'l2'], 'C':[0.1, 0.5, 1, 2,5] | C= 0.5, penalty=l2 |
| **SVM** | C':[0.001,0.005,0.01,0.015,0.02,0.1,0.2,,0.5,1,2,5],'gamma':[0.0001,0.001,0.002,0.005,0.01,0.015,0.02,0.1,1,10] | C=10, gamma=0.001 |
| **NB** | alpha': [0.1, 0.2, 0.5, 1, 2, 5] | alpha=0.1 |
| **XGBoost** | n_estimators':range(50,2000,10),'learning_rate':np.arange(0.01,1,0.01),'max_depth':range(1,10,1),'min_child_weight':range(1,10,1), 'gamma':[i/10.0for i in range(0, 5)], 'subsample':[i/10.0 for i in range(1, 10)],'colsample_bytree': [i/10.0 for i in range  (1,10)], 'scale_pos_weight':[i for i in range(1,10,1)] | n_estimators=500,  learning_rate=0.01, max_depth=4,  gamma=0.,  subsample=1,  colsample_btree=1,  scale_pos_weight=1 |
| **RF** | n_estimators':range(100,250,10),'max_depth':range(1,10,1),'min_samples_split':range(2,20,2),'min_samples_leaf':range(2,20,2),'max_features':range(3,11,2) | n_estimators=200,  max_depth=4,  min_samples_split=2,  min_samples_leaf=14,  max_features=7 |

LR, Logistic Regression; SVM, Support Vector Machines with Linear Kernel; NB, Naive Bayes; XGBoost, Extreme Gradient Boosting algorithm; RF, Random Forest.

**Supplementary Figure**

**Supplementary Figure 1. Time to CVD following index delivery.**

**References**

1. Le Cessie S, van Houwelingen JC. Ridge estimators in logistic regression. *Applied Statistics* (1992)41:191–201

2. Boser B. E., Guyon I. M., Vapnik V. N. A training algorithm for optimal margin classifiers. Proceedings of the 5th Annual ACM Workshop on Computational Learning Theory. Pittsburgh, Pa, USA.(1992). p. 144–152

3. Estimating Continuous Distributions in Bayesian Classifiers. Available at: https://arxiv.org/pdf/ 1302.4964. [Accessed December 15, 2018]

4. Breiman L. Random forests. *Mach Learn* (2001)45:5–32

5. Chen T, Guestrin, C. Xgboost: A scalable tree boosting system. In: Proceedings of the 22Nd ACM SIGKDD International Conference on Knowledge Discovery and Data Mining. (2016) ACM
