# Supplementary figures and images for: A Machine Learning-Based Prediction Model for Cardiovascular Risk in Women With Preeclampsia
Source: Front Cardiovasc Med. 2021 Oct 27;8:736491. doi: 10.3389/fcvm.2021.736491 (PMC8578855; doi:10.3389/fcvm.2021.736491)

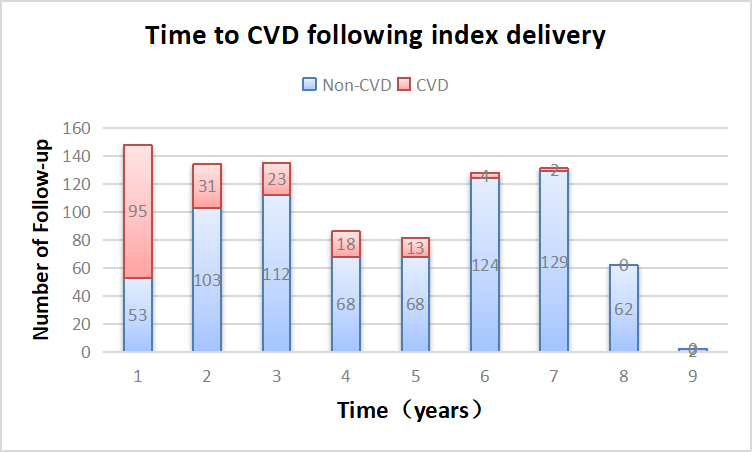

Supplement: Supplementary file 1 [file Presentation_1.zip › Supplementary_Material/Supplementary Figure 1.tif]
